# Electron extraction layer-driven performance enhancement in CaHfSe_3_ photovoltaics

**DOI:** 10.1039/d5ra05879a

**Published:** 2025-09-29

**Authors:** Hicham El-assib, Mohamed Alla, Safae Tourougui, Mustafa K. A. Mohammed, Shazia Akhtar Dar, Yasmine Labghough, Malika Alla, Naima, Mustapha Rouchdi, Boubker Fares

**Affiliations:** a (STCE)-Energy Research Centre (ERC), Faculty of Science, Mohammed V University Rabat Morocco; b MANAPSE Lab, Faculty of Sciences, Mohammed V University in Rabat Morocco; c College of Remote Sensing and Geophysics, Al-Karkh University of Science Baghdad 10011 Iraq dr.mustafa@ks.edu.iq; d College of Science, University of Warith Al-Anbiyaa Karbala 56001 Iraq; e Department of Electronics and Communication Engineering, National Institute of Technology Srinagar 190006 India; f Laboratory of Information Processing (LTI), Faculty of Sciences Ben MSick, University Hassan II Casablanca Morocco; g Department of Applied Physics, Delhi Technological Technology Delhi 110042 India

## Abstract

Traditional solar cells – including those based on silicon or lead-halide perovskites – have a number of significant disadvantages, including long-term instability, costs, and toxicity. We demonstrate the suitability of CaHfSe_3_ as a promising next-generation lead-free thermally stable absorber material. We performed a comprehensive numerical simulation study using SCAPS-1D to consider several device topologies of the type FTO/TiO_2_ AZnO, WS_2_/CaHfSe_3_/MoO_3_/Au, and to investigate the different features of a system based on CaHfSe_3_. We conducted a full parametric study of the impacts of absorber thickness, defect density, acceptor doping and concentration, as well as carrier concentrations in the electron and hole transport layers. In addition, through experimentation we considered the operational characteristics of carrier generation-recombination methods, temperature and back contact effect current–voltage *I*–*V* characteristics, quantum efficiency, and the influence of series and shunt resistance. This allowed us to determine the optimized configuration. The top-performing structure, FTO/TiO_2_/CaHfSe_3_/MoO_3_/Au, had an outstanding PCE of 32.39%, *V*_OC_ = 1.52 V, *J*_SC_ = 23.17 mA cm^−2^, and FF = 91.41%. This research offers both fundamental insights and practical guidance for developing stable, efficient, and environmentally friendly CaHfSe_3_-based solar cells. It paves the path for further experimental realization and commercial application.

## Abstract 2

Traditional solar cells – including those based on silicon or lead-halide perovskites – have a number of significant disadvantages, including long-term instability, costs, and toxicity.

## Graphical abstract

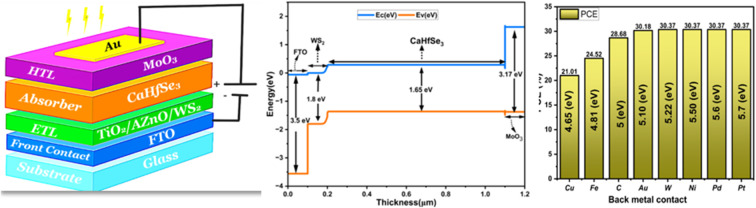

## 1. Introduction

Transition to renewable energy sources has emerged as a global imperative to address the challenges posed by climate change and rising electricity demand. Among energy conversion technologies, solar cells have experienced significant growth thanks to advances in developing new semiconductor materials.^1,2^ Although silicon-based photovoltaic cells currently dominate the market, they have certain limitations regarding manufacturing cost, efficiency, and stability. In particular, crystalline silicon suffers from intrinsic constraints as an indirect band gap semiconductor, its low absorption coefficient requires a minimum thickness to ensure sufficient light harvesting. Still, increasing thickness inevitably raises the series resistance.^3^ This trade-off between absorption and Joule losses fundamentally restricts the overall performance of silicon solar cells. These constraints have driven the exploration of alternative materials, especially perovskites, which are garnering increasing attention in this context due to their exceptional optical and electronic properties, cost-effectiveness, and sustainability for photovoltaic devices.^4–6^ Specifically, MAPbX_3_ or FABX_3_ lead halide perovskites have demonstrated remarkable power conversion efficiency (PCE) of 27%.^7^ However, conventional PSCs are weak in resilience to heat and humidity, have a high concentration of defects, are unstable due to the instability of organic cations, and contain poisonous lead (Pb), all of which pose significant health and environmental dangers, preventing their widespread commercialization. The primary obstacle is the search for lead (Pb)-free, stable, and eco-friendly materials. This is achieved by replacing cations such as Ge^2+^ or Sn^2+^, which have advantageous band gaps, for Pb^2+^ ions.^8,9^ However, the resultant perovskites are extremely unstable in air because of the strong oxidation propensity of Ge^2+^ and Sn^2+^ to Sn^4+^ and Ge^4+^, respectively, which is linked to the increased energy levels of their respective 5s and 4s orbitals.^10^ However, a major obstacle still stands in the way of these promising solar materials, poor durability.

This study is aimed at identifying materials with the positive attributes of lead halide perovskites while eliminating their disadvantages. Chalcogenide perovskites are one of the most promising alternatives due to their non-toxic nature, impressive structural stability, and good electronic properties. These compounds typically adopt the ABX_3_ structural formula, where A is an alkaline earth metal, B is a transition metal, and X is either sulfur (S) or selenium (Se).^11^ A broad range of ABX_3_ chalcogenide perovskites was recognized by Sun *et al.* in 2015 as potential absorber materials for solar applications. BaZrS_3_, CaZrS_3_, SrZrS_3_, BaHfS_3_, CaHfS_3_, SrHfS_3_, CaTiS_3_, BaZrSe_3_, CaZrSe_3_, SrZrSe_3_, BaHfSe_3_, SrHfSe_3_, and CaTiSe_3_ are some of the more prominent instances.^12^ Simulations highlight the strong potential of ABX_3_-based solar cells, but experimental realization is limited by the complexity of film growth and ambient stability issues, as defects and exposure to moisture or oxygen can reduce efficiency. Overcoming these challenges is essential to achieve the predicted performance. Crystalline chalcogenide perovskite (CP) thin films are typically synthesized at high temperatures, greater than 600 °C (1170 F), severely limiting the ability to fabricate both single and multi-junction solar cells. The production of high-quality CP films *via* physical deposition methods or chemical sulfidation processes typically involves temperatures as high as 700 to 1000 °C (1292 to 1832 F), where intermediate products frequently include impurity phases in the final composition.^13^ Additionally, fabricating CP thin films *via* solution-based methods remains a significant challenge, as no known solvent can withstand the extreme temperatures needed for their synthesis. Furthermore, in 2025, Hayes *et al.* devised a comprehensive method for synthesizing various ABS_3_ compounds, such as BaZrS_3_, BaTiS_3_, SrZrS_3_, and SrHfS_3_, together with colloidal phases synthesized within the Ba–Hf–S chemical system. These materials are composed of elements with a strong affinity for oxygen, notably group IV transition metals like Ti, Zr, and Hf.^14^ This study identifies deficiencies in current research, noting that while BaZrS_3_ nanoparticles have been manufactured in various phases by colloidal techniques, a consistent procedure for orthorhombic perovskite nanoparticles has not been established. To solve this, the authors introduce a high-temperature hot-injection technique, which allows precise control over the formation of colloidal BaZrS_3_ nanoparticles, leading to a more reliable and scalable synthesis process. Recent studies on the ABS_3_ chalcogenide perovskite show that replacing S with Se dramatically lowers the bandgap moving it away from the visible to the near-infrared wavelength. This transformation improves the capability for photovoltaic implementation and appear more favorable for transitioning solar energy technology.^15^ Hf based perovskites still suffer from bulk recombination mainly due to Hf vacancy defects.^16^ The lead-free perovskite CaHfSe_3_ emerges as a viable alternative, as it combines the advantageous characteristics of semiconductors with properties comparable to its lead-based counterparts. These include favorable charge-carrier effective masses, a direct and optimal band gap, high absorption coefficients, and exceptional thermal stability, making it a promising absorber material for next-generation photovoltaics. The optical spectrum of the CaHfSe_3_ reports a clear absorption edge which corresponds to a direct electronic bandgap of 1.65 eV.^15^ This optimization study of CaHfSe_3_-based perovskite solar cells (PSCs) focuses on various combinations of electron and hole transport layers (ETL–HTL), using SCAPS-1D simulations, a one-dimensional solar cell modelling software based on capacitance. To enhance device performance, MoO_3_ is employed as the HTL in combination with TiO_2_, WS_2_, and AZnO as ETL candidates. Key operational parameters such as back contact material, series resistance, and operating temperature are systematically examined. Additionally, the effects of absorber layer thickness and doping concentration are optimized to further improve cell efficiency. Analysis of current–voltage (*J*–*V*) characteristics and external quantum efficiency (EQE) confirms that the optimized configuration yields a significantly enhanced power conversion efficiency (PCE) compared to previously reported designs.

## 2. Device architecture and computational methodology

### 2.1. Numerical modeling technique

The numerical method employed in this research is based on simulations conducted with SCAPS-1D 3.3.11 (Solar Cell Capacitance Simulator – 1D), a widely used software for modeling the electrical behavior of thin-film solar cells, which solves the coupled Poisson's and continuity equations under steady-state conditions. As shown in eqn (1) Poisson equation, relates electrostatic potential to charge density in the material within the device:^17^1
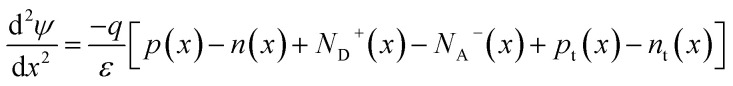
With *ε*: dielectric constant, *q*: electron charge, *ψ*: electrostatic potential, *n*, and *p*: free electrons and holes, *n*_t_ and *n*_p_: trapped electrons and holes, *N*_D_^+^and *N*_A_^−^: represent the densities of ionized donors and acceptors, respectively.

Poisson's equation must be solved in order to analyze solar cells since it shows how the electric potential is dispersed throughout the device's many layers. This knowledge facilitates the analysis of charge carrier dynamics, which involves the movement and recombination of electrons and holes to generate energy. The continuity equations reflect the principle of charge conservation and describe the motion of charge carriers in semiconductors. In this case, eqn (2) and (3) represent continuity equations for electrons and holes, respectively.2
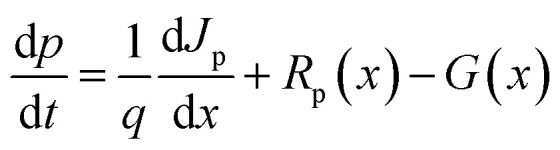
3
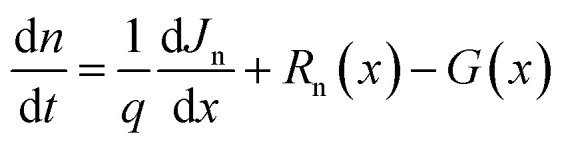
where *G* is the photogeneration rate, *R*_n_ and *R*_p_ are the rates of recombination of electrons and holes, respectively and *J*_n_ and *J*_p_ being the electrical current densities of electrons and holes. In semiconductor materials, electrons or holes can be migrating for two reasons—there is an electric field that produces a drift current or there is a concentration gradient of carriers so that there is a diffusion current. Eqn (4) and (5) describe the electrical current densities associated with electron and hole transport.4
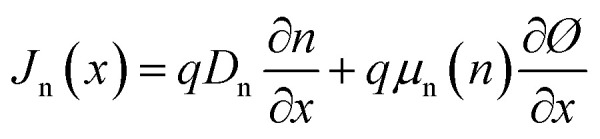
5
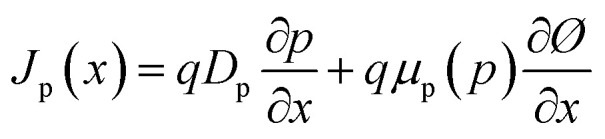


Using the optical absorption model, the optical absorption constant *α* is computed, as shown in eqn (6).6
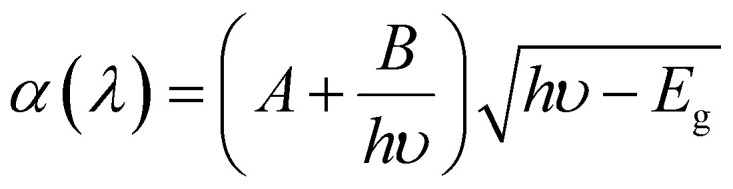
where *υ* is the radiation frequency, *E*_g_ is the material's real band gap, *h* is Planck's constant, and *A* and *B* are the model parameters.

### 2.2. Simulation settings and device structure

**Fig. 1:**
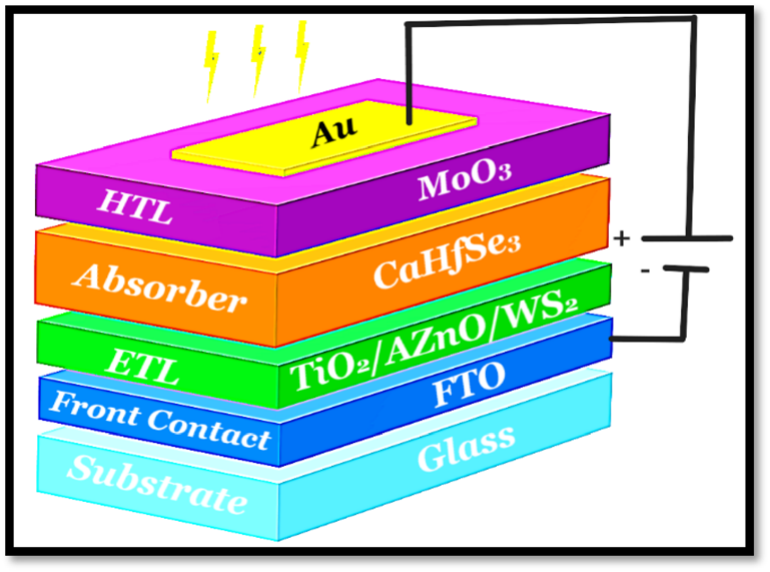
The device architecture of the PSC based on CaHfSe_3_.

In the current work, SCAPS-1D Software was used to model a device with the structure of (back metals/MoO_3_/CaHfSe_3_/ETMs/FTO), as shown generally in Fig. 1, to simulate the one-dimensional performance of a thin films photovoltaic device. The ETMs WS_2_, AZnO, and TiO_2_ were used due to their properties that complement the structural and electronic fabric of the ETLs for improved extraction of electrons through the layers to the collecting circuits of solar cell architectures. The two different ETLs are used along with MoO_3_ as a hole transport layer or HTL layer and the studies explored the pairs of combinations that produced the best configuration for the device. The materials used for the CaHfSe_3_ absorber layer, the FTO-coated glasses, which are transparent conducting materials, and the back contact varied with Cu, Fe, Au, C, Ni, and Pt. The project is focused on improving the metrics and performance of CaHfSe_3_-based lead-free perovskite solar cells by simulating modifications to the HTLs (WS_2_, AZnO, TiO_2_), and ETL(MoO_3_), and the back contacts (Cu, Fe, Au, C, Ni, Pt). For each layer, key parameters were defined, such as thickness, bandgap energy, electron affinity, permittivity, doping concentration, carrier mobilities, and defect densities, are listed in Table 1.

**Table 1:** The key input values defining the architecture of the proposed solar cell

Parameters	FTO^18^	TiO_2_ (ref. 16)	CaHfSe_3_ (ref. 19)	WS_2_ (ref. 20)	MoO_3_ (ref. 21)	AZnO^8^
Thickness (μm)	0.1	0.03	Varied	0.03	0.03	0.03
Bandgap *E*_g_ (eV)	3.5	3.33	1.65	1.8	3.17	3.33
Electron affinity *χ* (eV)	4	4.55	4	3.95	2.05	4.55
Dielectric permittivity (relative)	9	8.12	7.93	13.6	12.5	8.12
*N*_c_ effective density of states in CB (cm^−3^)	2.2 × 10^18^	4.1 × 10^18^	8.9 × 10^18^	1 × 10^18^	2.21 × 10^19^	4.1 × 10^18^
*N*_v_ effective density of states in VB (cm^−3^)	1.8 × 10^19^	8.2 × 10^19^	1.1 × 10^19^	2.4 × 10^19^	1.8 × 10^19^	8.2 × 10^19^
*V*_th,e_ velocity of electrons (cm s^−1^)	1 × 10^7^	1 × 10^7^	1 × 10^7^	1 × 10^7^	1 × 10^7^	1 × 10^7^
*V*_th,p_ velocity of holes (cm s^−1^)	1 × 10^7^	1 × 10^7^	1 × 10^7^	1 × 10^7^	1 × 10^7^	1 × 10^7^
*μ*_n_ mobility of electrons (cm^2^ V^−1^ s^−1^)	20	100	18.77	100	25	100
*μ*_p_ mobility of holes (cm^2^ V^−1^ s^−1^)	10	20	6.81	100	100	20
*N*_D_ shallow uniform donor density (cm^−3^)	21 × 10^19^	11 × 10^13^	0	1 × 10^18^	0	11 × 10^13^
*N*_A_ shallow uniform acceptor density (cm^−3^)	0	0	1 × 10^19^	0	1 × 10^19^	0
*N*_t_ density of defect (cm^−3^)	1 × 10^15^	1 × 10^17^	1 × 10^13^	1 × 10^15^	1 × 10^15^	1 × 10^17^

Table 2 presents the work functions of various materials, including Gold (Au), Nickel (Ni), Carbon (C), Iron (Fe), Copper (Cu), and Platinum (Pt). The simulations were conducted under standard AM1.5G illumination (1000 W m^−2^) at an operating temperature of 300 K. Additionally, series/shunt resistances and working temperature were incorporated to reflect realistic electrical behavior. Device performance was assessed using *J*–*V* characteristics, quantum efficiency (QE), and power conversion efficiency (PCE), with systematic variation of key parameters to determine the optimal configuration.

**Table 2:** The contact work functions applied in the configurations

Metal contacts	Work function (eV) (ref. 8 and 18)
Cu	4.65
Fe	4.81
C	5
Au	5.1
Ni	5.5
Pt	5.7

## 3. Outcome analysis and interpretation

### 3.1. The band diagram of the model cell

**Fig. 2:**
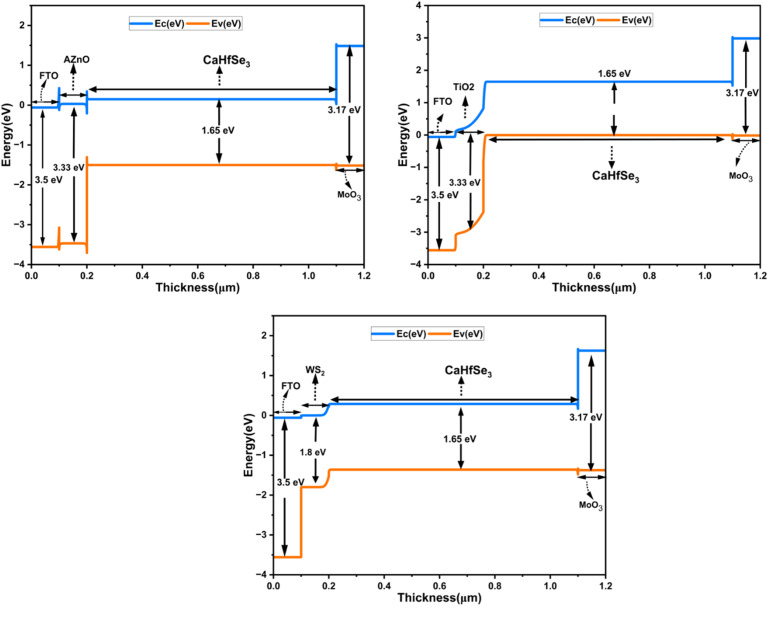
CaHfSe_3_-based PSC energy diagram with MoO_3_ as the HTL and (a) AZnO, (b) TiO_2_, and (c) WS_2_ as the ETL.

Simulated band diagrams for the proposed structures are shown in Fig. 2, highlighting the importance of energy level alignment in achieving efficient charge separation and high photovoltaic performance. To ensure effective electron transport, the electron affinity of the electron transport layer (ETLs) must exceed that of the CaHfSe_3_ absorber, facilitating downward conduction band alignment.^22^ Conversely, hole extraction is enhanced when the ionization potential of the hole transport layer (MoO_3_) is lower than that of the absorber, ensuring a smooth flow of holes.^23^

The WS_2_/CaHfSe_3_ interface exhibits a small CBO (0.02 eV), indicating good energy level continuity and promoting efficient electron transport. In the case of TiO_2_, which has a wide *E*_g_ of 3.33 eV, the conduction band alignment (0.01 eV) also supports efficient electron extraction while acting as a barrier to hole backflow. The AZnO/CaHfSe_3_ interface shows a conduction band offset of approximately 0.03 eV, reflecting strong energetic compatibility and potential for high electron collection efficiency. On the hole transport side, the CaHfSe_3_/MoO_3_ interface demonstrates a favorable valence band offset (VBO), as the valence band maximum (VBM) of MoO_3_ lies below that of CaHfSe_3_. This ensures selective and efficient hole extraction while suppressing electron recombination at the back contact. Altogether, these band alignments confirm the suitability of WS_2_, TiO_2_, and AZnO as ETLs, and MoO_3_ as an HTL, in facilitating efficient charge separation and transport in CaHfSe_3_-based photovoltaic devices.

### 3.2. Coefficient of absorption

**Fig. 3:**
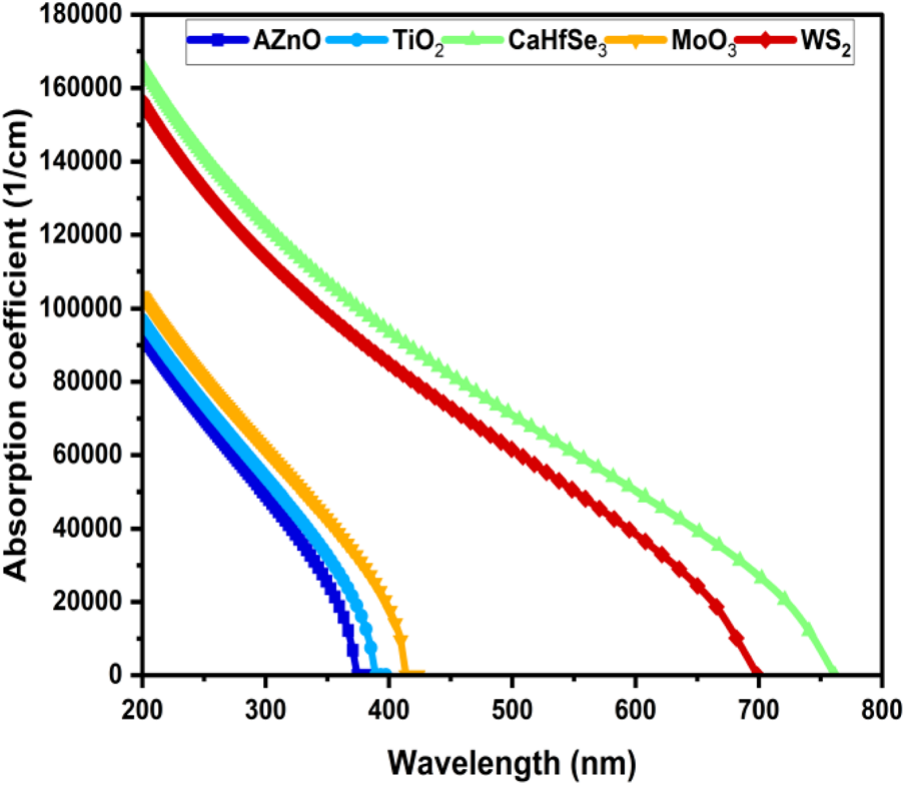
Absorption coefficients of CaHfSe_3_, TiO_2_, AZnO, WS_2_, and MoO_3_ as a function of wavelength.

The coefficient of absorption (*α*) as a function of wavelength indicates a material's ability to absorb light across various regions of the electromagnetic spectrum (UV, visible, and infrared). Fig. 3 displays the theoretical absorption profiles for all layers. The absorbent material CaHfSe_3_ is notable for its strong Capability of absorbing radiation in the visible region, with values exceeding 10^5^ cm^−1^ between 400 and 770 nm. This characteristic makes it an excellent choice for harnessing solar light and producing charge carriers. TiO_2_ and AZnO, used as electron transport layers (ETL), exhibit very low absorption coefficients (less than 10^2^ cm^−1^ between 350 nm and 700 nm), allowing light to pass through with minimal absorption and facilitating optimal transmission to the active CaHfSe_3_ layer. Finally, MoO_3_, used as a hole transport layer (HTL), demonstrates intermediate behavior with a low absorption coefficient (around 10^3^) in the 350–700 nm range, minimizing interaction with incident light. Its absorption increases slightly in the UV range but remains compatible with its role.^24^

### 3.3. Impact of ETL and absorber thickness on device performance

**Fig. 4:**
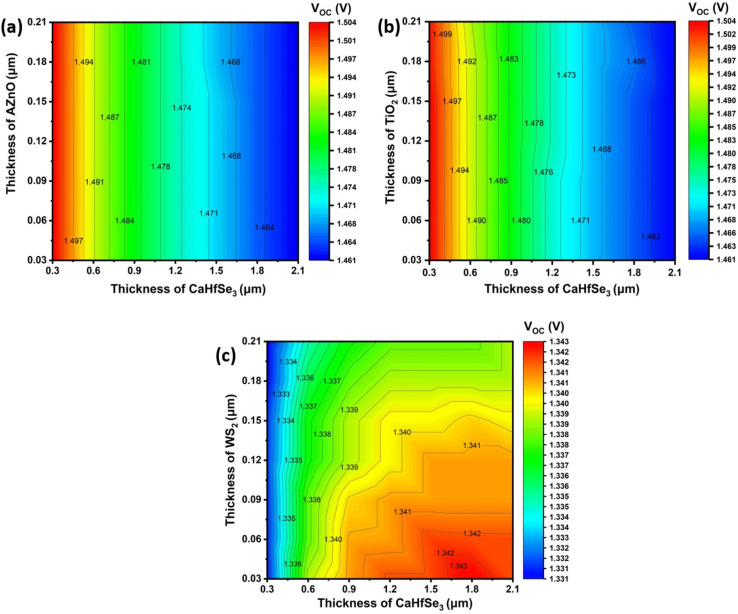
*V*_OC_ contour mapping for (a) AZnO, (b) TiO_2_, and (c) WS_2_ ETLs with CaHfSe_3_ absorber thickness.

To improve light harvesting and promote efficient extraction of charge carriers, the absorption and ETL thicknesses must be carefully chosen and optimized. Maximum photon absorption in the active layer and efficient electron transport are ensured by a well-optimized design. In this study, contour plots were used to investigate the impact of absorber and ETL thickness variations on the performance of CaHfSe_3_ architectural performance has been investigated. Through this research, we can investigate how altering the thickness of the absorber and electron transport layers affects key performance metrics, including *V*_OC_, FF, *J*_SC_, and PCE. To identify the optimal thickness combinations for enhanced device performance, the contour plots display the predicted values of *J*_SC_, *V*_OC_, FF, and PCE across a range of absorber thicknesses (300 to 2100 nm) and ETL thicknesses (from 30 to 210 nm). In this investigation section, MoO_3_ was held constant as the HTM. Fig. 4 shows the impact of absorber layer thickness and ETL on the *V*_OC_. Under conditions where the absorber and ETL layers were about 300 nm and <150 nm in thickness, respectively, the perovskite employing TiO_2_ and AZnO as ETLs reached a peak *V*_OC_ of 1.54 V, as illustrated in Fig. 4(a and b). As the thicknesses of both the absorber and ETL layers grew, the open-circuit voltage (*V*_OC_) exhibited a decline. This decrease can be attributed to a rise in the reverse saturation current caused by the thicker absorber, combined with the partial absorption of incident light within the thicker ETL layers. By contrast, when absorber and ETL thicknesses were approximately 1500 nm and ≤30 nm, respectively, the PSC with a WS_2_ ETL showed a maximum *V*_OC_ of 1.34 V.

**Fig. 5:**
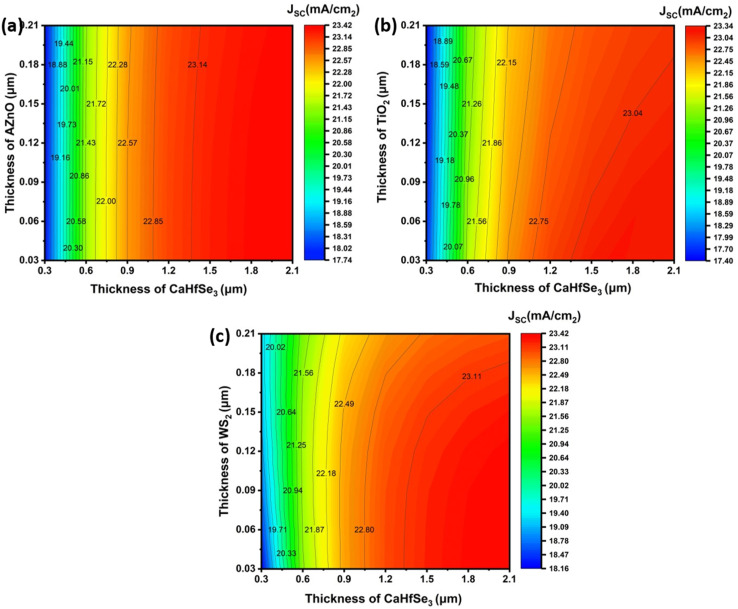
*J*_SC_ contour mapping for (a) AZnO, (b) TiO_2_, and (c) WS_2_ electron transport layers (ETLs) as a function of CaHfSe_3_ absorber thickness.

**Fig. 6:**
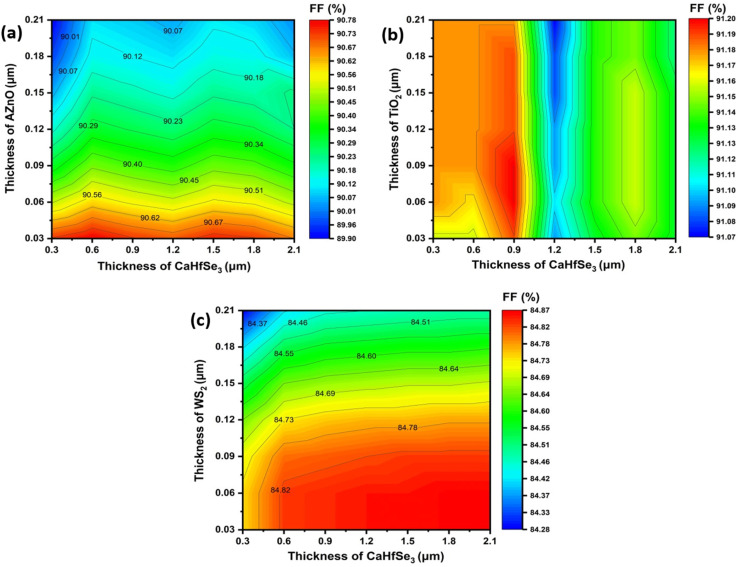
Contour mapping of the fill factor (FF %) for different ETL materials: (a) AZnO, (b) TiO_2_, and (c) WS_2_.

The influence of changes in the absorber layer thickness and ETL on the *J*_SC_ parameter of three selected perovskite solar cells is illustrated in Fig. 5. The PSC incorporating TiO_2_ as the ETL achieved a maximum current density of 23.33 mA cm^−2^ once the absorber layer exceeded 1800 nm and the ETL thickness was ≥30 nm. Under conditions where the absorber measured 2100 nm and the thickness of ETL ranged from 30 to 210 nm, the ETL AZnO demonstrated the greatest *J*_SC_ of 23.4 mA cm^−2^. In addition, the PSCs using WS2 ETL exhibited maximum (*J*_SC_) of 23.4 mA cm^−2^ when the absorber thickness was 2100 nm and the ETL thickness ranged from 30 to 90 nm. Generally, an increase in absorber thickness led to higher *J*_SC_ values in each device, as a thicker absorber enhances photon absorption, promotes greater electron–hole pair generation, and consequently boosts the photocurrent. This enhancement is also ascribed to the improved spectral responsiveness at extended wavelengths. Fig. 6 illustrates how different absorber and ETL layer thicknesses affect FF values. When WS_2_ is implemented as the electron transport layer (ETL) in solar cell structures, studies have revealed that the FF of BaHfSe_3_-based solar cells increase with absorber thickness up to 1.8–2.1 μm, while it slightly decreases as the WS_2_ layer becomes thicker. The highest FF value of 84.86% is recorded when the BaHfSe_3_ thickness ranges from 1.8 to 2.1 μm and the WS_2_ thickness is between 0.03 and 0.06 μm, Fig. 6(b).

**Fig. 7:**
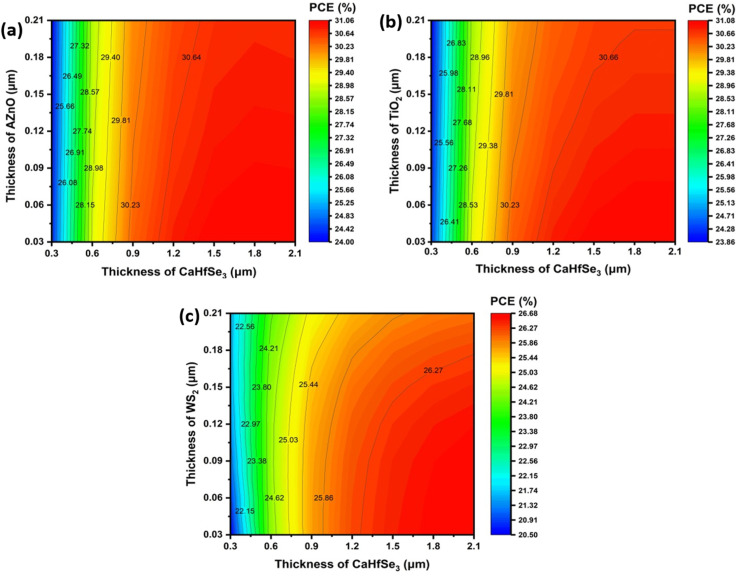
Contour mapping of PCE (%) when ETL is (a) AZnO, (b) TiO_2_, and (c) WS2.

Using TiO_2_ as an ETL results in a maximum fill factor (FF) of 91.2% with a BaHfSe_3_ thickness of 0.9 μm and a TiO_2_ layer thickness ranging from 0.06 to 0.09 μm. In contrast, increasing the thickness of BaHfSe_3_ beyond 1.2 μm leads to a slight decline in FF, likely resulting from enhanced recombination losses or reduced efficiency in charge transport. Furthermore, Fig. 6(a) illustrates that the AZnO electron transport layer thickness significantly impacts the fill factor (FF), with ultrathin layers (≤0.03 μm) showing optimal values (∼90.7%). FF gradually decreases as AZnO thickness increases, primarily due to heightened recombination losses and series resistance. Conversely, variations in the BaHfSe_3_ absorber's thickness have minimal effect on the FF, indicating consistent device performance over a wide thickness range (0.3–2.1 μm), as charge carrier extraction and production remain unchanged. Fig. 7 displays the simulation findings showing distinct performance characteristics for the three ETLs when incorporated into BaHfSe_3_-based solar cells. AZnO-based devices reach a top PCE of 31.05% when configured with a 1.8 μm absorber and a 30 nm ETL, showcasing a favorable trade-off between efficiency and material economy. Notably, AZnO maintains good efficiency at reduced absorber thicknesses, making it a low-cost and easy-to-fabricate alternative. However, the devices with TiO_2_ exhibit competitive performance, achieving a slightly higher maximum PCE of 31.08% with a thicker absorber layer of 2.1 μm. Its reliance on increased thickness implies a greater dependence on photon absorption to compensate for its reduced electron mobility. Conversely, WS_2_ shows a lower overall efficiency, with a maximum PCE of 26.67% occurring at WS_2_ thicknesses less than 0.15 μm combined with absorber thicknesses ranging from 1.8 to 2.1 μm.

There are several reasons for the noticeable differences in *V*_OC_, *J*_SC_, FF, and PCE across perovskite with various ETL. The absorption coefficient is a crucial component that greatly impacts photovoltaic metrics, including *V*_OC_, *J*_SC_, FF, and PCE. This parameter affects the coupling efficiency of light photons with the underlying CaHfSe_3_ absorber layer and is directly correlated with the ETL's bandgap. The overall efficiency of the solar cell is significantly affected by the CBO, arising from the disparity in electron affinity and Fermi level positions between the absorber and the ETL.

### 3.4. Effect of absorber acceptor concentration and layer thickness on photovoltaic performance

**Fig. 8:**
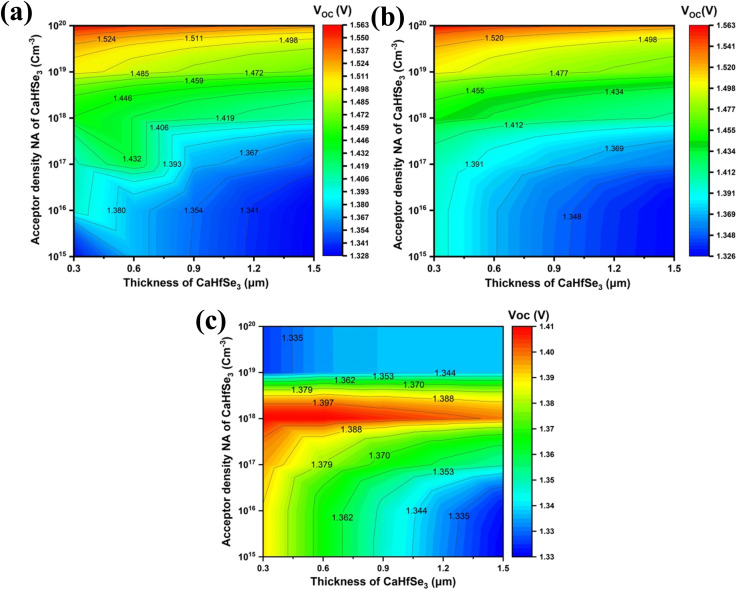
Contour plots illustrate the influence of absorber doping concentration and thickness on *V*_OC_ for ETL types (a) AZnO, (b) TiO_2_, and (c) WS_2_.

Optimizing both the absorber thickness and *N*_A_ within the absorber is essential for achieving improved efficiency. To examine the impact of these factors, we conducted simulations by adjusting the acceptor doping density (*N*_A_) from 1 × 10^15^ to 1 × 10^20^ cm^−3^ and absorber thickness from 0.3 to 1.5 μm across seven distinct PSC configurations. Fig. 8 shows how variations in absorber thickness and *N*_A_ influence the (*V*_OC_) in the validated PSC configurations.

According to Fig. 8, under conditions where the absorber was less than 0.9 μm thick and the acceptor doping concentration (*N*_A_) was fixed at 10^20^ cm^−3^, AZnO and TiO_2_ achieved their maximum *V*_OC_ values of 1.5 V. Notably, increasing the absorber thickness beyond this threshold resulted in a gradual decline in *V*_OC_ for all ETLs under the same doping conditions. The data's shape makes it evident that while *V*_OC_ tends to drop with increasing absorber thickness, it increases with increased acceptor concentration (*N*_A_). This behavior is explained by the stronger electric field and greater built-in potential at higher doping levels, which makes it easier to separate and remove photogenerated carriers and increase *V*_OC_. Nevertheless, the series resistance rises, and the greater sheet resistance at thicker absorber layers constrains the hole mobility towards the HTL. This leads to a decrease in carrier collection and, thus, a lower *V*_OC_.^25^

**Fig. 9:**
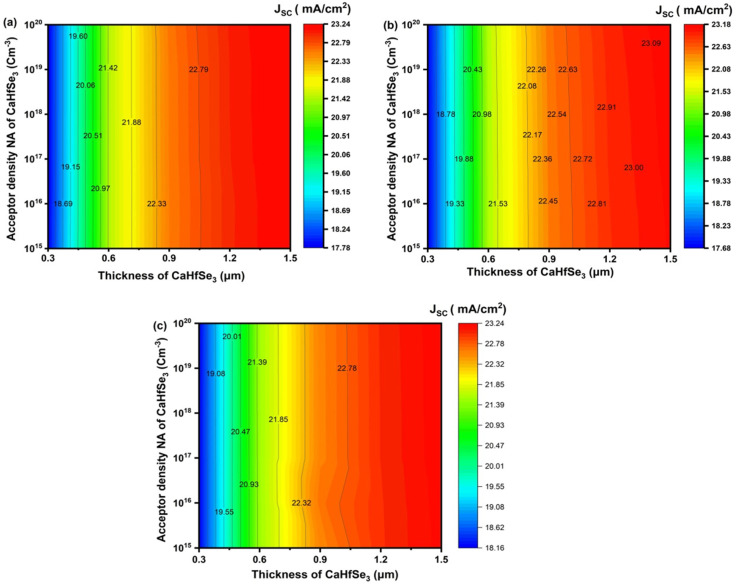
Contour plots illustrate the influence of absorber doping concentration and thickness on *J*_SC_ for ETL types (a) AZnO, (b) TiO_2_, and (c) WS_2_.

**Fig. 10:**
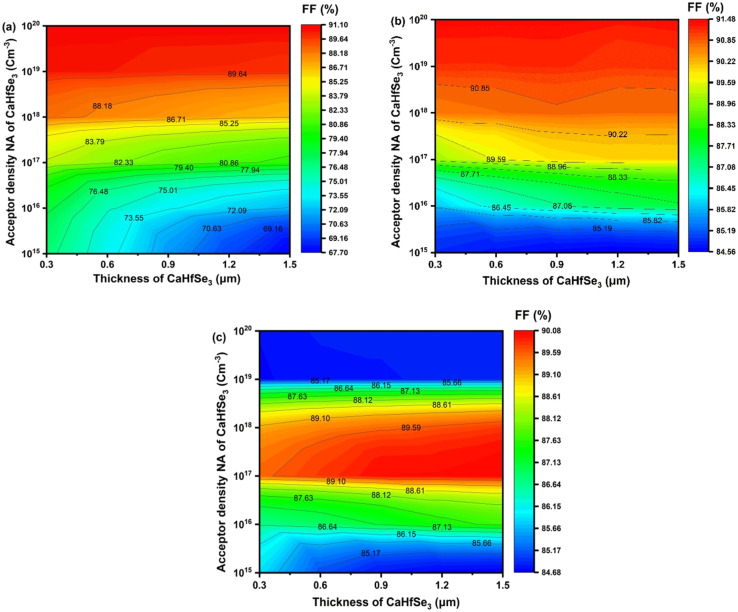
Impact of (a) AZnO, (b) TiO_2_, and (c) WS_2_ ETLs on FF (%) contour plots in CaHfSe_3_ perovskite solar cells.

Fig. 9(a)–(c) show that the short-circuit current density *J*_SC_ for devices using ZnO, TiO_2_, and WS_2_ as ETL shows a significant increase with absorber thickness and a modest increase with increasing acceptor density (*N*_A_). The maximum *J*_SC_ values of 23.239 mA cm^−2^, 23.169 mA cm^−2^ and 23.232 mA cm^−2^ for ZnO, TiO_2,_ and WS_2_, respectively, were observed at an absorber thickness of 1.5 μm with *N*_A_ = 10^20^ cm^−3^. The increased light absorption in the thicker CaHfSe_3_ absorber layer, which permits a bigger production of photogenerated carriers, is primarily responsible for the progressive increase in *J*_SC_ seen for all ETLs from 0.3 μm to 1.5 μm. Furthermore, for a constant thickness, the *J*_SC_ values are rather stable over *N*_A_ values and improve slightly with increasing *N*_A_. This is explained by the improved separation of photogenerated carriers in highly doped materials, leading to more effective electric field generation and less recombination. Fig. 10(a)–(c) illustrate that, for all three ETLs, the fill factor (FF) typically increases with the rising acceptor density (*N*_A_) throughout all examined thicknesses.

This enhancement results from minimized carrier recombination and enhanced charge transport as a result of increased doping levels. TiO_2_ often demonstrates the highest fill factor values, achieving 91.48% at a minimal thickness of 0.3 μm and elevated doping levels (*N*_A_ > 10^19^ cm^−3^). This exceptional performance is attributed to its effective surface passivation characteristics and favorable band alignment with CaHfSe_3_, which reduces non-radiative recombination. At low acceptor densities, AZnO exhibits lower FF values than TiO_2_, but, when *N*_A_ rises, it shows notable improvement, reaching up to 91.03–91.06% for *N*_A_ = 10^20^ cm^−3^. However, we observe that the WS_2_-based solar cell structure exhibits a maximum FF value, reaching nearly 90% when the thickness of absorber exceeds 0.8 μm, and *N*_A_ ranges from 10^17^ to 10^18^ cm^−3^.

**Fig. 11:**
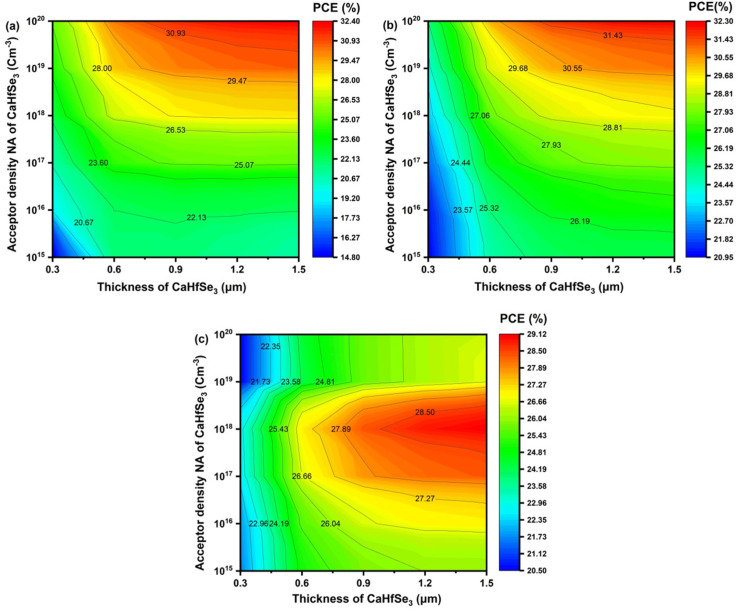
PCE (%) contour plots with ETL as (a)AZnO, (b) TiO_2_, and (c) WS_2_.

Fig. 11(a) and (b) highlight the combined effect of absorber thickness and *N*_A_ on the performance of CaHfSe_3_-based solar cells. The simulations show that the PCE reaches its peak at absorber thicknesses of 1.2 μm and 1.5 μm, achieving 32.30% for AZnO and 32.40% for TiO_2_ when *N*_A_ exceeds 10^19^ cm^−3^. A reduction in absorber thickness leads to a noticeable drop in PCE, since thinner layers allow a considerable fraction of incident photons to pass through unabsorbed, thereby limiting electron–hole pair generation. Conversely, thicker absorbers enhance light absorption, increase carrier generation, and result in higher efficiencies. In addition, the PCE improves with increasing *N*_A_, as higher acceptor doping concentrations strengthen the built-in electric field, which facilitates the separation and transport of photogenerated carriers, reduces recombination losses, and ultimately enhances device performance.^26^Fig. 11(c) shows that using WS_2_ as the ETL results in a PCE of 29.12% when the absorber thickness exceeds 1.2 μm and the *N*_A_ is approximately 10^18^ cm^−3^. This phenomenon occurs because a high acceptor density increases the recombination rate, restricting carrier mobility and subsequently reducing the PCE.

### 3.5. Influence of defect density and CaHfSe_3_ layer thickness on photovoltaic performance

Defect density is a crucial factor that significantly impacts perovskite solar cells (PSCs). Absorber layer defects, acting as recombination sites, reduce carrier lifetimes and decrease device efficiency. Consequently, achieving excellent performance necessitates reducing the absorber layer's defect density. Numerous studies have focused on the interplay between defect density and absorber thickness in CaHfSe_3_-based PSCs and their effect on device efficiency and the results demonstrate a significant relationship between the two factors. Optimizing both simultaneously can result in the most efficient device possible.

**Fig. 12:**
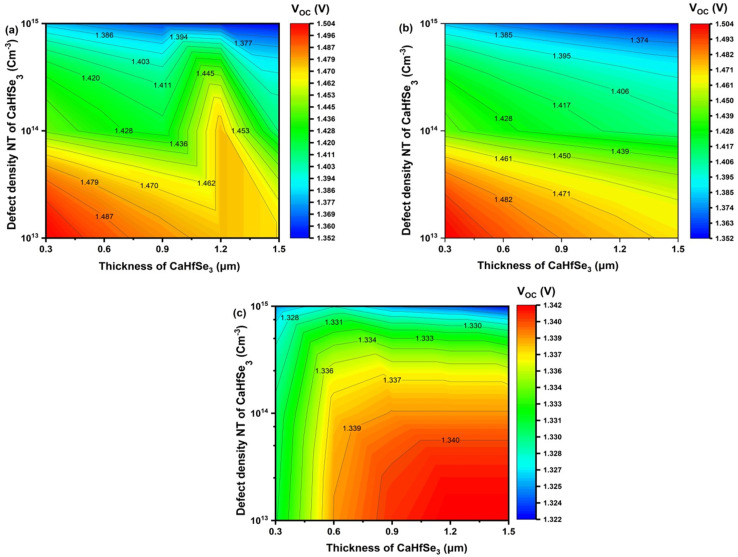
Contour graphs of *V*_OC_ dependence on CaHfSe_3_ absorber thickness and defect density with ETLs as (a) AZnO, (b) TiO_2_, and (c) WS_2_.

Hence, the thickness of the CaHfSe_3_ absorber ranged from 300 nm to 1500 nm, alongside *N*_t_ values adjusted from 10^13^ to 10^15^ cm^−3^. The simulation outcomes indicate that both the absorber thickness and *N*_t_ substantially affect the *V*_OC_ of CaHfSe_3_-based solar cells, as shown in Fig. 12. For all evaluated ETLs (AZnO, TiO_2_, and WS_2_), In configurations (a) and (b), *V*_OC_ exhibits a strong dependence on both thickness and defect density. When *N*_t_ is maintained below 10^14^ cm^−3^, *V*_OC_ reaches values as high as 1.448 V, indicating minimal nonradiative recombination and efficient carrier extraction. The voltage increases with thickness up to approximately 0.9 μm, beyond which the gain plateaus, suggesting that additional thickness does not significantly enhance photogeneration or reduce recombination. This behavior reflects a balance between optical absorption and carrier diffusion length, where excessive thickness may introduce resistive losses or trap-limited transport without further improving voltage. As *N*_t_ increases beyond 10^16^ cm^−3^, a pronounced decline in *V*_OC_ is observed, regardless of thickness. This trend highlights the detrimental impact of bulk and interfacial defects, which act as recombination centers and suppress quasi-Fermi level splitting. The steep voltage gradient across the defect density axis underscores the necessity of defect passivation strategies, such as surface treatments or compositional tuning, to preserve high *V*_OC_ values. In contrast, configuration (c) yields significantly lower *V*_OC_ values, ranging from 1.128 V to 1.148 V. The voltage remains relatively insensitive to thickness variations, and even at low *N*_t_, the performance is markedly inferior to that of configurations (a) and (b). This suggests a fundamentally different device architecture or material interface, potentially characterized by poor band alignment, high interface recombination velocity, or inadequate carrier selectivity. The suppressed *V*_OC_ may also reflect unfavorable energetics at the contact layers or the presence of deep-level traps within the CaHfS_3_ matrix. Particularly at low defect concentrations (*N*_t_ = 10^13^ cm^−3^), since the longer carrier transport pathways raise the possibility of recombination. Furthermore, the greatest *V*_OC_ values reported are 1.504 V for both AZnO and TiO_2_ at 0.3 μm thickness and *N*_t_ = 10^13^ cm^−3^, and 1.342 V for WS_2_ at 1.2–1.5 μm thickness with the same low defect density.

**Fig. 13:**
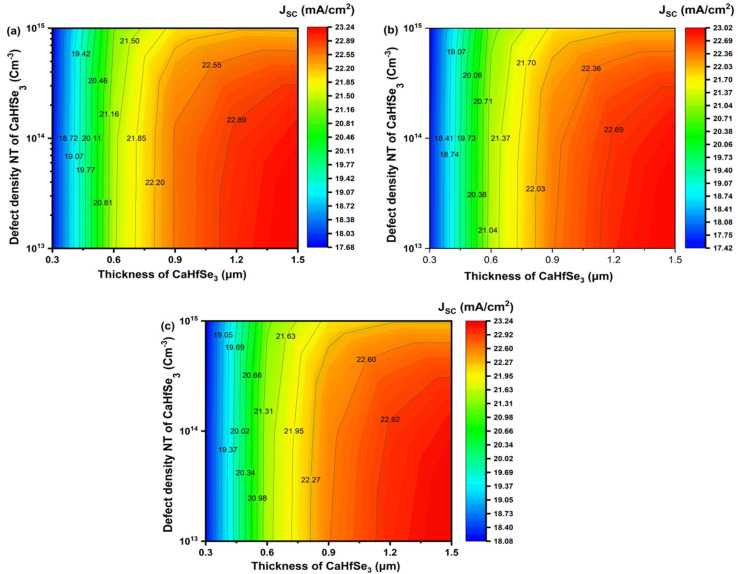
Contour graphs of *J*_SC_ as a function of CaHfSe_3_ absorber thickness and defect density with ETL as (a) AZnO, (b) TiO_2_, and (c) WS_2_.

Fig. 13 shows that the *J*_SC_ of CaHfSe_3_-based solar cells is significantly influenced by both the absorber thickness and *N*_t_. Because larger layers generate more carriers and absorb more photons, *J*_SC_ grows continuously with absorber thickness. Beyond 1.2 μm, this gain tends to saturate, suggesting an ideal thickness range for balancing recombination and absorption losses. On the other hand, *J*_SC_ slightly decreases when the defect density is increased from 10^13^ to 10^15^ cm^−3^ at a given thickness. This is because greater trap-assisted recombination lowers the carrier collection efficiency. The maximum *J*_SC_ values recorded are 23.24, 23.02, and 23.24 mA cm^−2^ for AZnO, TiO_2_, and WS_2_, respectively. These values all correspond to a low defect density <10^14^ cm^−3^ and an absorber thickness greater than 1.2 μm. These results validate that adequate absorber thickness and defect passivation are both necessary to maximize photo-generated current.

**Fig. 14:**
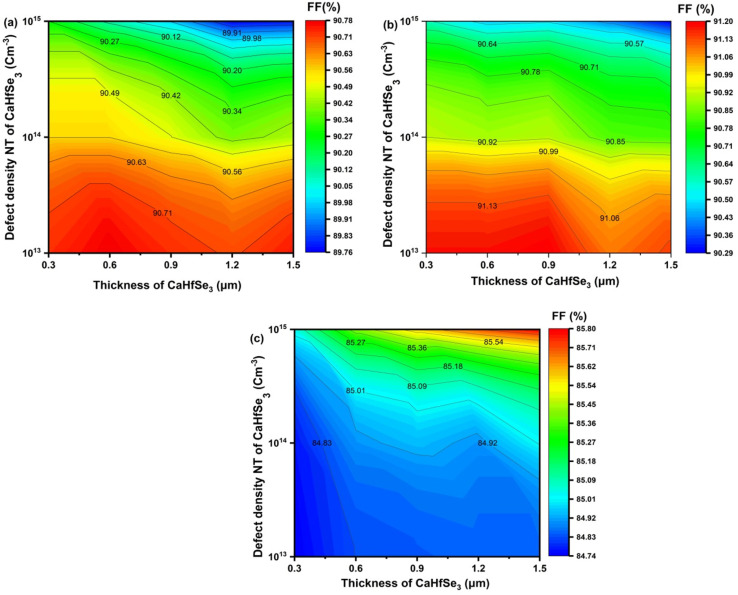
Contour graphs of FF as a function of CaHfSe_3_ absorber thickness and defect density with ETL as (a) AZnO, (b) TiO_2_, and (c) WS_2_.

For the device utilizing AZnO as the electron transport layer, Fig. 14(a), the fill factor exhibited considerable stability across varying absorber thicknesses and defect concentrations. In particular, FF values were higher than 90.50% for thicknesses <900 nm and *N*_t_ < 10^15^ cm^−3^. The highest FF of 90.78% was observed at *N*_t_ = 10^13^ cm^−3^ and thickness ∼0.6 μm. At 1.2 μm thickness, FF decreased from 90.78% to 89.76% as *N*_t_ grew from 10^13^ to 10^15^ cm^−3^, indicating a modest drop with rising *N*_t_. Due to accumulated recombination. Thus, low *N*_t_ and thickness allow for better fill factor, especially internal losses. Furthermore, the TiO_2_-based ETL device exhibited steady performance over diverse absorber thickness and *N*_t_ conditions, as presented in Fig. 14(b). A maximum fill factor (FF) of 91.20% was achieved at *N*_t_ = 1.0 × 10^13^ cm^−3^ and a thickness of 0.9 μm. As the defect density increased, the FF gradually declined; however, it remained above 90% under all tested conditions. This highlights the excellent compatibility of TiO_2_ with the CaHfSe_3_ absorber layer, as well as its effectiveness in minimizing series resistance and enabling efficient charge extraction.

In contrast, the device employing WS_2_ as the ETL displayed a lower but increasing FF trend with absorber thickness. Fig. 14(c) indicates that FF rose from 85.04% to 85.80% as thickness increased from 0.3 μm to 1.5 μm at *N*_t_ = 1.0 × 10^15^ cm^−3^, achieving a maximum FF of 85.80%. At low *N*_t_ values (<10^14^ cm^−3^), FF stayed below 85.00%, suggesting that WS_2_ is more suitable for thicker absorbers with higher defect tolerance. The lowest result, 84.74%, was recorded at 0.3 μm and *N*_t_ = 1.0 × 10^13^ cm^−3^.

**Fig. 15:**
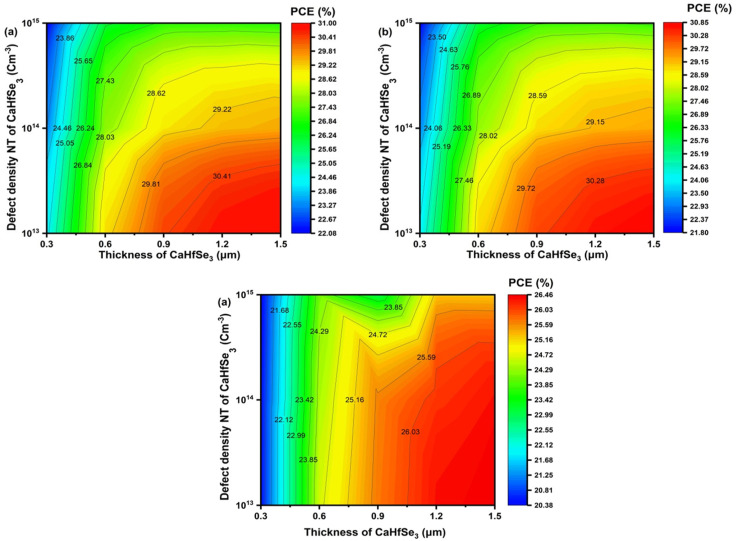
PCE contour maps showing the influence of CaHfSe_3_ absorber thickness and defect density for ETLs including (a) AZnO, (b) TiO_2_, and (c) WS_2_.

Fig. 15 shows the PCE of CaHfSe_3_-based solar cells utilizing three different ETLs: AZnO, TiO_2_, and WS_2_, with various absorber thicknesses and defect densities. For all ETLs, the PCE increased with absorber thickness and dropped with increasing defect density (*N*_t_), which corresponded to improved light absorption and lower carrier recombination at low *N*_t_. The device achieved a maximum PCE of 31.00% when AZnO served as the ETL, combined with a carrier concentration of 10^13^ cm^−3^ and an absorber thickness of 1.5 μm. Moreover, Fig. 15(b) shows that the device using TiO_2_ as the ETL demonstrated high performance, achieving a maximum PCE of 30.81% under similar conditions. In contrast, Fig. 15(c) reveals that WS_2_ exhibited a lower peak efficiency of 26.46% at *N*_t_ equals 1.0 × 10^13^ cm^−3^ and an absorber thickness of 1.5 μm. While both AZnO and TiO_2_ showed high and comparable efficiencies across all examined conditions, WS_2_ consistently delivered lower efficiencies, particularly at low thicknesses and high defect densities. This behavior may be attributed to increased interfacial recombination or higher series resistance. These findings underscore the excellent performance and compatibility of AZnO and TiO_2_ as ETLs for CaHfSe_3_-based devices, particularly when combined with low defect densities and optimized absorber thickness.

### 3.6. Effect of HTM and ETM thickness and carrier concentration on PV properties

#### 3.6.1. Effect of HTM thickness

**Fig. 16:**
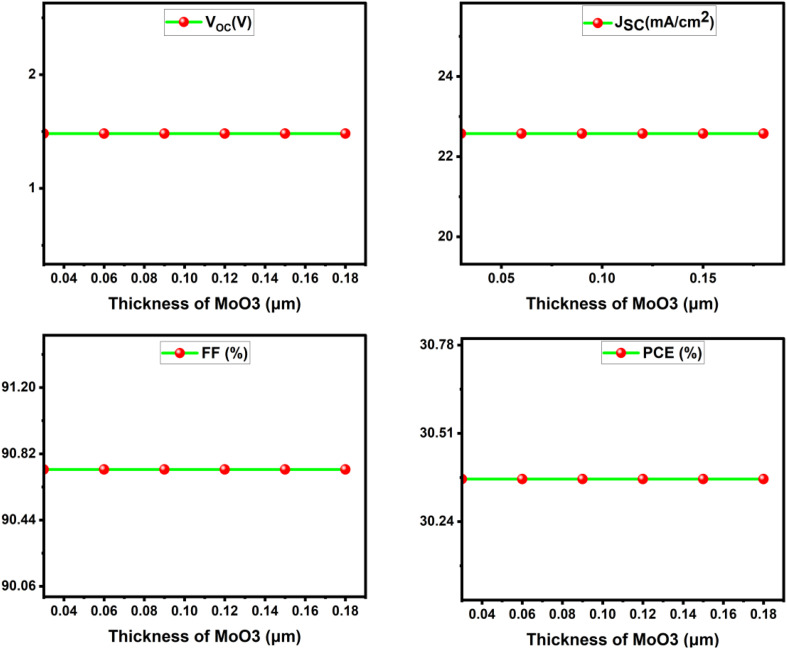
Influence of MoO_3_ layer thickness on the solar cell photovoltaic characteristics.

**Fig. 17:**
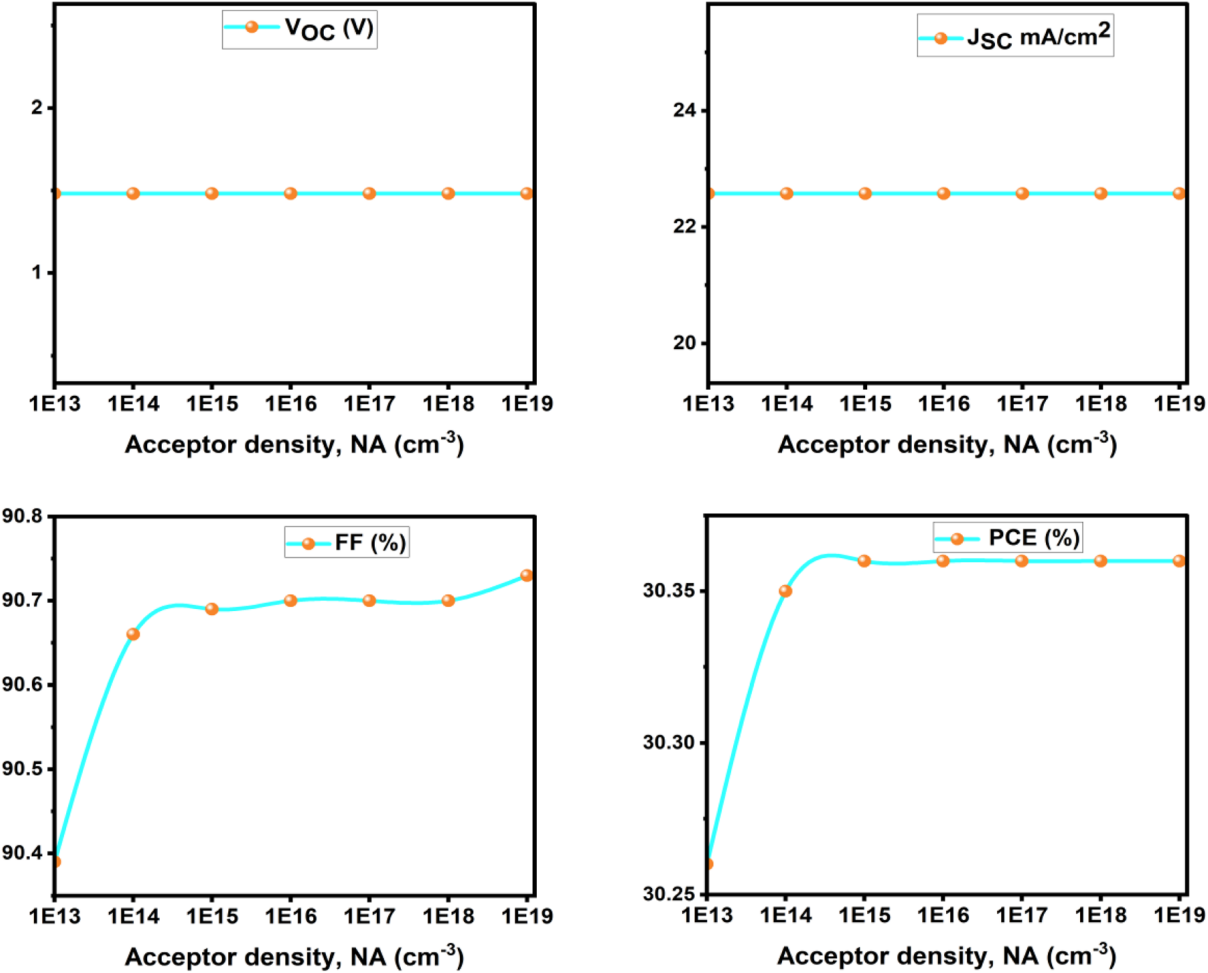
Effect of MoO_3_ acceptor doping concentration (*N*_A_) on photovoltaic parameters.

The HTL improves efficiency by collecting holes, restricting electron movement, and shielding the perovskite from environmental variables including moisture, heat, and oxygen.^27^ This section uses AZnO as the ETM and MoO_3_ as the HTM. HTL thickness was varied from 20–180 nm to evaluate how thickness influences the performance of a CaHfSe_3_-based PSC. Fig. 16 shows that all the significant metrics for performance, namely *V*_OC_, *J*_SC_, FF and PCE, were not significantly affected over the range of thicknesses and showed only slight differences in overall performance. Specifically, *V*_OC_ = 1.482 V, *J*_SC_ = 22.575 mA cm^−2^, FF = 90.73% and PCE = 30.37%. Overall, the MoO_3_ layer provided a suitable energy level for hole transport and sufficient holes across the range of HTL thickness without adding series resistance, or introducing any optical losses. Even the lowest thickness was suitable in ensuring effective hole extraction and hole transport (30 nm), as evidenced from the data showing that the performance metrics were not generally influenced by HTL thickness. Therefore, reduced HTL thickness is a solution to cut material usage or production cost while remaining similar in efficacy (Fig. 17).

#### 3.6.2. Impact of the concentration of HTM carriers

The results indicate that *V*_OC_ (1.482 V) and *J*_SC_ (22.575 mA cm^−2^) remain almost unchanged with increasing *N*_A_. This stability arises because these parameters are primarily governed by the absorber's intrinsic optoelectronic properties, including bandgap, absorption coefficient, and carrier lifetime which are not significantly affected by moderate changes in the MoO_3_ HTL doping level. Consequently, carrier generation, separation, and transport within the absorber remain stable, in agreement with previous simulation studies on the limited effect of HTL doping on *V*_OC_ and *J*_SC_. However, the FF shows a slight improvement, rising from 90.39% to 90.73% as *N*_A_ increases. The improvement is probably caused by increased hole transport efficiency and reduced series resistance at the HTL, owing to higher doping levels, enabling better charge extraction. The PCE rises from 30.26% to 30.36%, plateauing above a doping concentration of 10^15^ cm^−3^.

#### 3.6.3. Impact of the concentration of ETM carriers

**Fig. 18:**
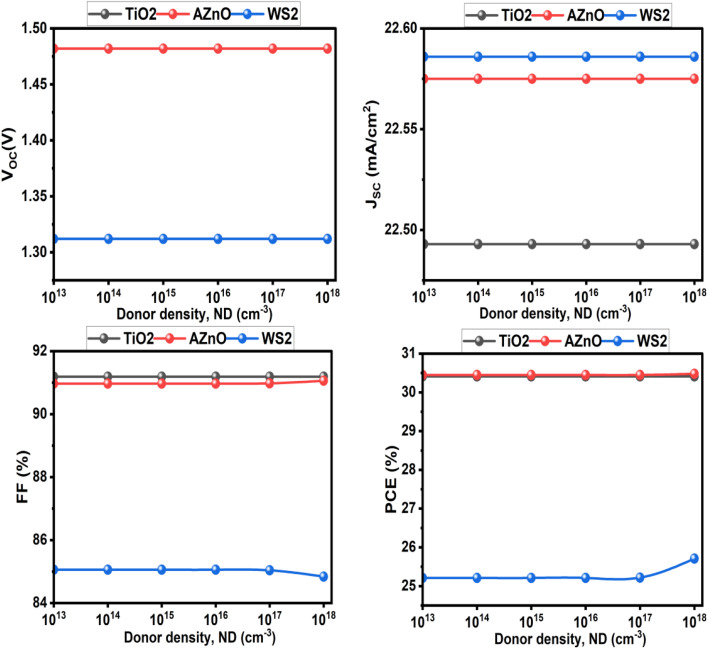
Effect of ETM donor doping concentration (*N*_D_) on photovoltaic parameters.

**Fig. 19:**
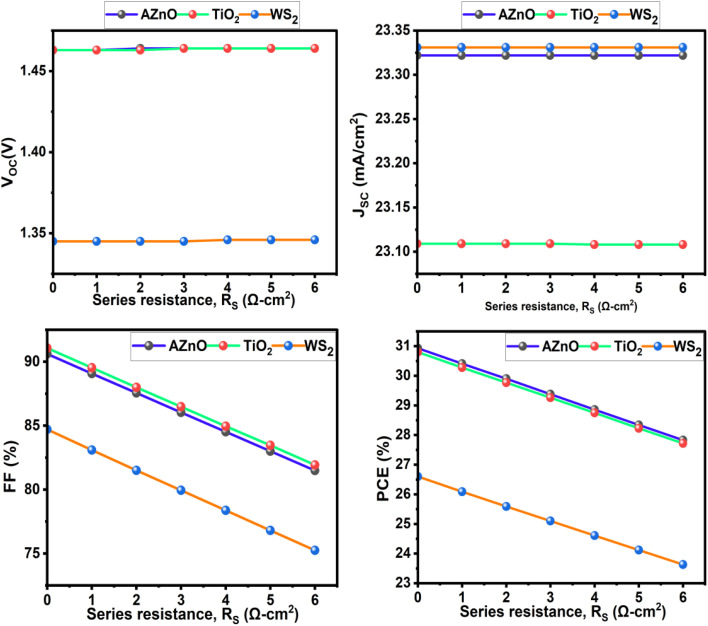
The impact of series resistance on solar cell performance under varied ETLs.

Fig. 18 illustrates the influence of the ETL donor doping density (*N*_D_) on the photovoltaic performance of solar cells utilizing TiO_2_, ZnO, and WS_2_. TiO_2_ demonstrates consistent characteristics at all doping levels, maintaining a steady PCE of 30.41%, thus affirming its durability. Furthermore, AZnO shows a slight improvement in fill factor and efficiency as *N*_D_ increases, achieving a maximum power conversion efficiency of 30.48%. However, WS_2_, with a maximum efficiency of 25.71%, demonstrates poorer and more doping-sensitive performance. In contrast *V*_OC_ is mainly determined by the absorber bandgap and the splitting of the quasi-Fermi levels, which are controlled by carrier generation and recombination within the absorber. Since ETL doping does not directly affect these intrinsic mechanisms, *V*_OC_ remains unchanged. Similarly, *J*_SC_ is governed by photon absorption and the diffusion/collection of photogenerated carriers in the absorber. ETL doping does not alter the absorption spectrum or the intrinsic carrier generation, so *J*_SC_ also stays constant (Fig. 19).

### 3.7. Impact of series resistance on performance

The series resistance (*R*_S_) in a solar cell generally arises from three sources: carrier transport within the emitter and base layers, contact resistance at the metal–semiconductor interfaces, and the resistance of the front and rear metallic contacts. Minimizing *R*_S_ is crucial, as high values cause internal voltage drops that reduce the fill factor (FF) and overall power conversion efficiency (PCE). Excessive resistive losses can significantly reduce the fill factor. In the current investigation, when *R*_S_ escalates from 0 to 6 Ω cm^2^, *V*_OC_ and *J*_SC_ stay rather stable throughout all ETLs, however, the FF and PCE diminish gradually. TiO_2_ and AZnO show excellent starting efficiencies of 30.93% and 30.80%, respectively, which decline to 27.83% and 27.72% at the maximum *R*_S_. WS_2_ starts at a lower PCE of 26.60%, dropping to 23.63%. This demonstrates that increasing series resistance affects device performance largely *via* lowering FF.

### 3.8. Impact of shunt resistance on performance

**Fig. 20:**
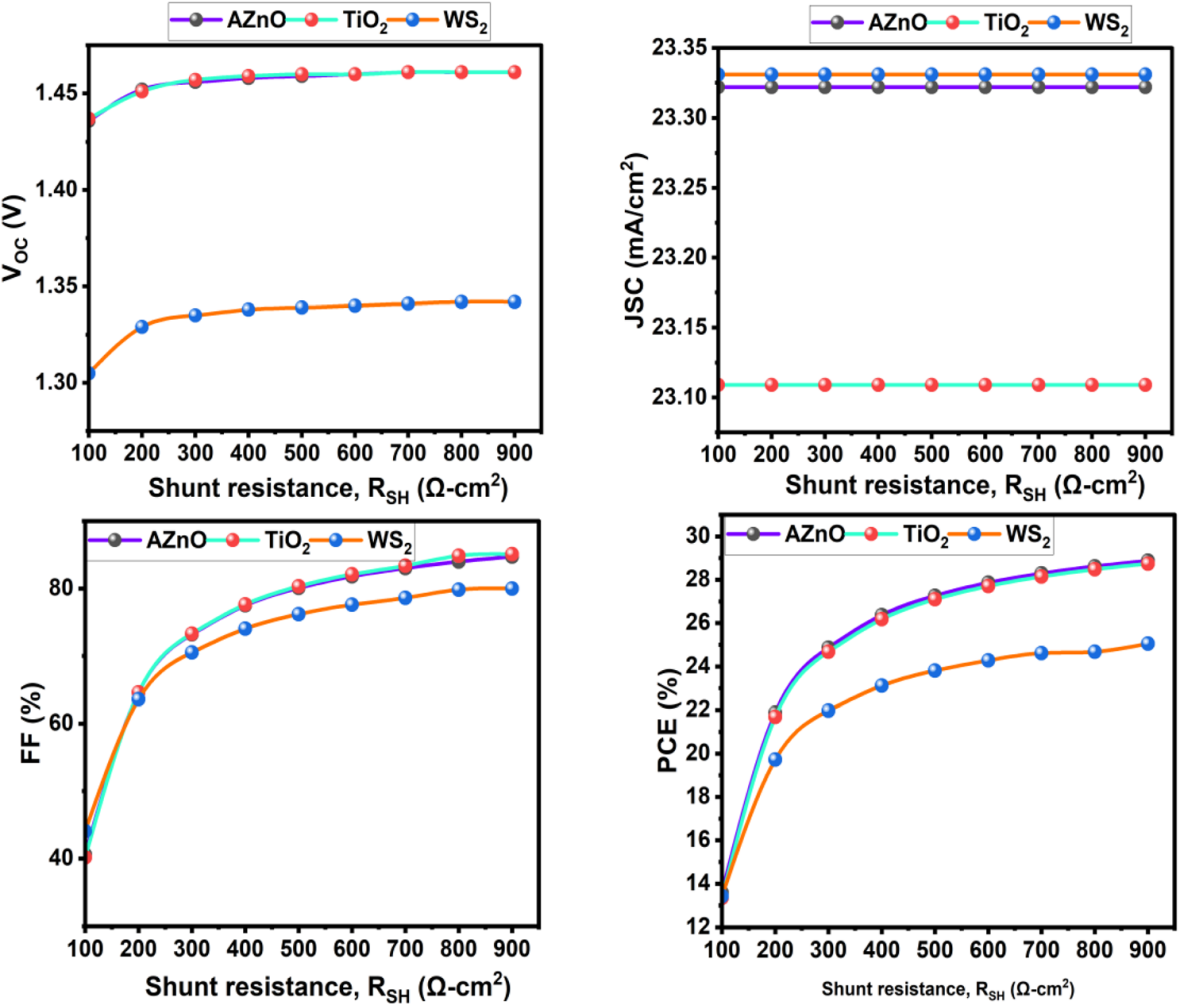
Effect of *R*_SH_ on the PSCs parameters.

The shunt resistance (*R*_SH_) corresponds to leakage pathways through defects, pinholes, or imperfect interfaces. A low *R*_SH_ leads to significant losses in open-circuit voltage (*V*_OC_) and a decrease in FF, whereas a high *R*_SH_ ensures that the majority of photogenerated carriers contribute to power output. The influence of shunt resistance (*R*_SH_) on the photovoltaic performance of solar cells using AZnO, TiO_2_, and WS_2_ as electron transport layers (ETLs) was studied. Fig. 20 shows that raising *R*_SH_ from 100 to 900 Ω cm^2^ significantly improved FF and PCE across all configurations. This behavior is explained by suppressing leakage currents, which are usually linked to manufacturing flaws, and avoiding the junction using low-resistance shunt routes. In particular, when *R*_SH_ rose, FF for the AZnO-based device improved from 40.59% to 84.71%, and PCE improved from 13.59% to 28.88%. At *R*_SH_ = 900 Ω cm^2^, the TiO_2_- and WS_2_-based cells showed comparable improvements, with final PCE values of 28.74% and 25.05%, respectively. The fill factor is the metric most sensitive to changes in shunt resistance, as seen by the relatively consistent *V*_OC_ and *J*_SC_ over the *R*_SH_ range.

### 3.9. Effect of temperature

**Fig. 21:**
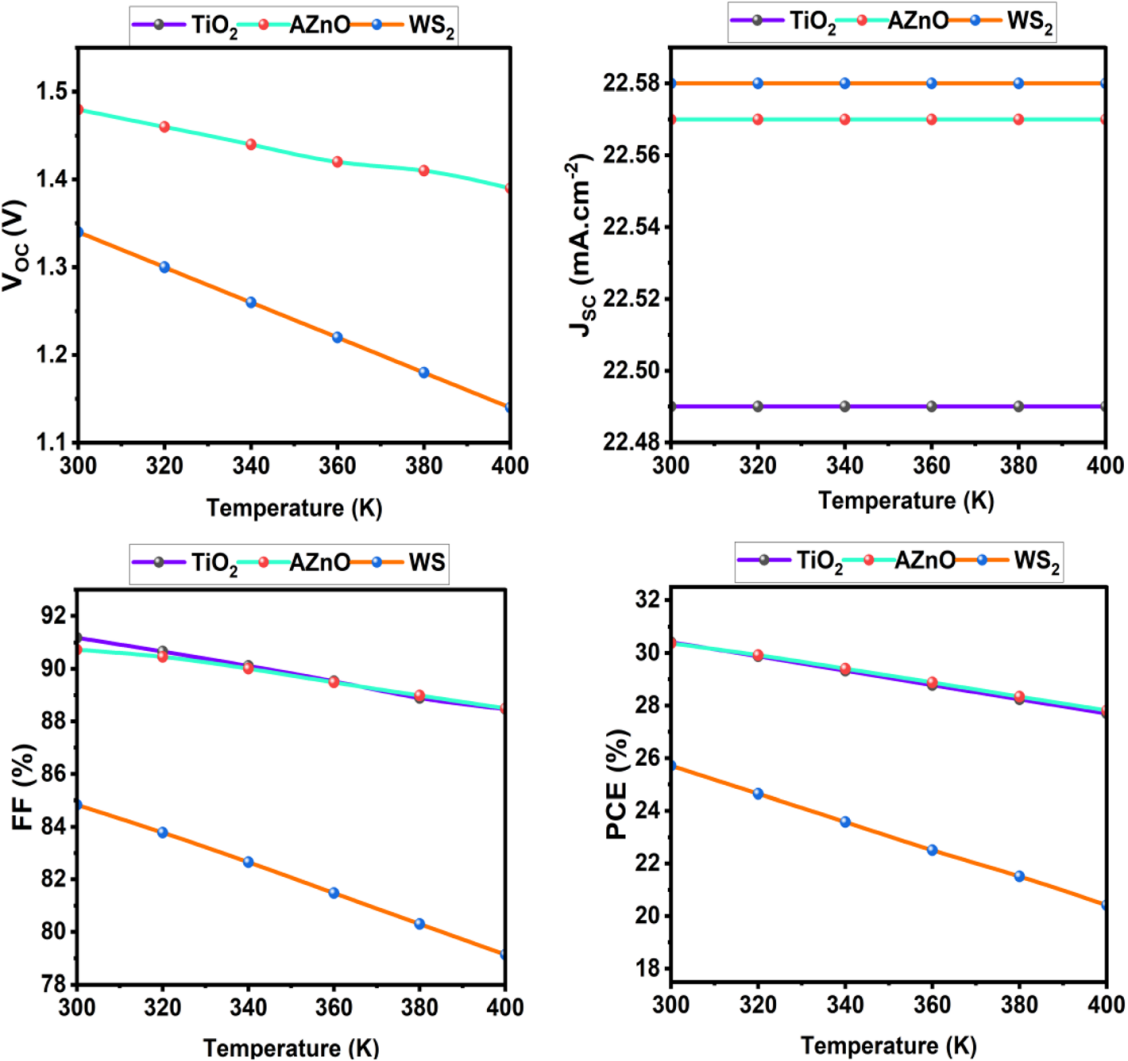
Impact of temperature on solar cell performance using TiO_2_, AZnO, and WS_2_ as ETLs.

One of the most difficult issues with PSC is guaranteeing its long-term stability, particularly at high temperatures. High temperatures can induce chemical and structural changes in perovskite materials, leading to a significant reduction in device performance. Furthermore, thermal stress may damage the integrity of the interfaces between distinct layers, causing greater charge carrier recombination and impeding charge transfer.^28^ To better understand the thermal response of PSC in actual working situations, a rigorous performance study was performed across a temperature range of 300 K to 400 K. Fig. 21. It has been demonstrated that increasing the temperature (from 300 to 400 K) reduces the *V*_OC_, fill factor (FF), and PCE, while the *J*_SC_ remains essentially constant. This drop is mostly because of more recombination and energy loss at high temperatures, making removing charges and lowering the potential difference across the cell. TiO_2_ and AZnO have similar performance, with *V*_OC_ values of 1.48 V, PCEs surpassing 30% at 300 K, and strong thermal stability. WS_2_ had lower results (*V*_OC_ of 1.34 V, PCE ∼25.7%) and more noticeable deterioration with temperature, indicating inefficient charge extraction and higher recombination. In contrast, the simulated *J*_SC_ remains nearly constant between 300 K and 400 K because photon absorption and carrier generation in the absorber are not significantly affected by temperature, while temperature mainly influences *V*_OC_ through the increase of the saturation current density.

### 3.10. Examination of the rates of recombination and generation

Elevating electrons from the B_V_ to the B_C_ causes holes to develop in the valence band, which in turn creates electron–hole pairs. This process is the essential mechanism for creating charge carriers in the substance. In SCAPS-1D, the generation rate *G*(*x*) is determined by the input photon flux *N*_phot_ (*λ*,*x*), which indicates the quantity of photons accessible for absorption at each depth *x* and wavelength *λ*. This photon flux is utilized to estimate the spatial generation profile within the absorber layer according to eqn (7):

7 *G*(*λ*,*x*) = *α*(*λ*, *x*)·*N*_phot_(*λ*, *x*)

**Fig. 22:**
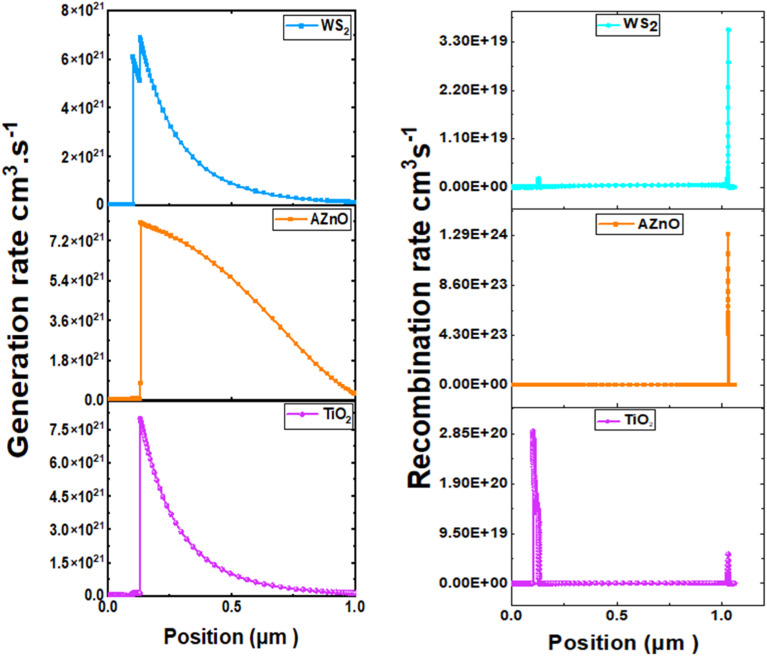
Generation and recombination profiles in CaHfSe_3_ absorber using TiO_2_, AZnO, and WS_2_ as ETLs.

It is essential to integrate insights from carrier generation-recombination mechanisms and band structure studies to optimize absorber thickness, doping levels, and interface engineering, while selecting suitable ETLs and HTLs. This comprehensive knowledge enhances the efficiency, stability, and overall performance of CaHfSe_3_-based solar cells. Fig. 22 illustrates carrier generation and recombination rates at various depths (0 to 1.1 μm) in three CaHfSe_3_-based CP solar cells. Investigations indicate that generation rates for all devices peak between 0.15 and 0.3 μm. In contrast, the recombination rate acts as a limiting factor, neutralizing these electron–hole pairs and preventing them from increasing the photocurrent. Carrier density and lifespan are the primary factors influencing the recombination rate. Moreover, defects present within the absorber and at its interfaces considerably increase the rate of electron–hole recombination. These imperfections, often caused by impurities, structural faults, or grain boundaries, lead to a non-uniform distribution of the recombination rate across the material. The PSC with WS_2_ and AZnO ETL had the greatest recombination rates in the 1 to 1.1 μm range, where AZnO as the ETL exhibited the highest rate. This occurs when additional electrons in the B_C_ bridge the *E*_g_ and join the B_V_, becoming stable and taking the position of a hole.

### 3.11. Back contact effect on PV parameters

**Fig. 23:**
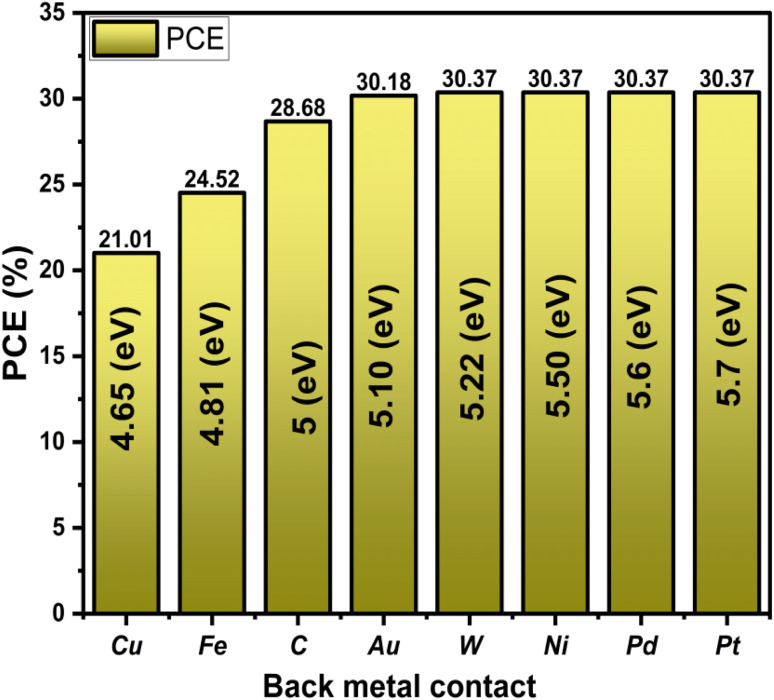
Influence of the back electrode work function on solar cell PCE.

Eight distinct metals were used as rear electrodes (Table 3). In simulations for structure based on FTO/AZnO/CaHfSe_3_/MoO_3_, exhibiting work function values spanning from 4.65 to 5.70 eV, to assess the impact of the back contact Work function on device performance. The results are summarized in Fig. 23. It has been discovered that the PCE improves with an increasing work function, eventually reaching a saturation point of roughly 5.2 eV. At lower levels, the reduced PCE results from creating a Schottky barrier at the metal/absorber interface, which prevents effective hole extraction and promotes interfacial recombination. Furthermore, as the work function exceeds ∼5.0 eV, the energy alignment at the back contact becomes more favorable for hole transport, lowering barrier height and improving carrier extraction. This leads to a fast rise in efficiency, reaching a maximum of around 30.37%. Beyond this level, the device's performance plateaus, indicating that the contact performs ohmically. Significantly, while gold ensures superior device performance, it accounts for around 20% of the overall production cost in perovskite solar cells. Electrodes composed of Ni, Pd, and Pt provide cost-effective alternatives, demonstrating promising efficiency levels that render them feasible substitutes for Au without significantly compromising device performance.

**Table 3:** The contacts used in the setups and their work function

Back electrode	Cu	Fe	C	Au	W	Ni	Pd	Pt
Work function (eV) (ref. 29)	4.65	4.82	5.00	5.30	5.22	5.50	5.60	5.70

### 3.12. Quantum efficiency and *J*–*V* properties

**Fig. 24:**
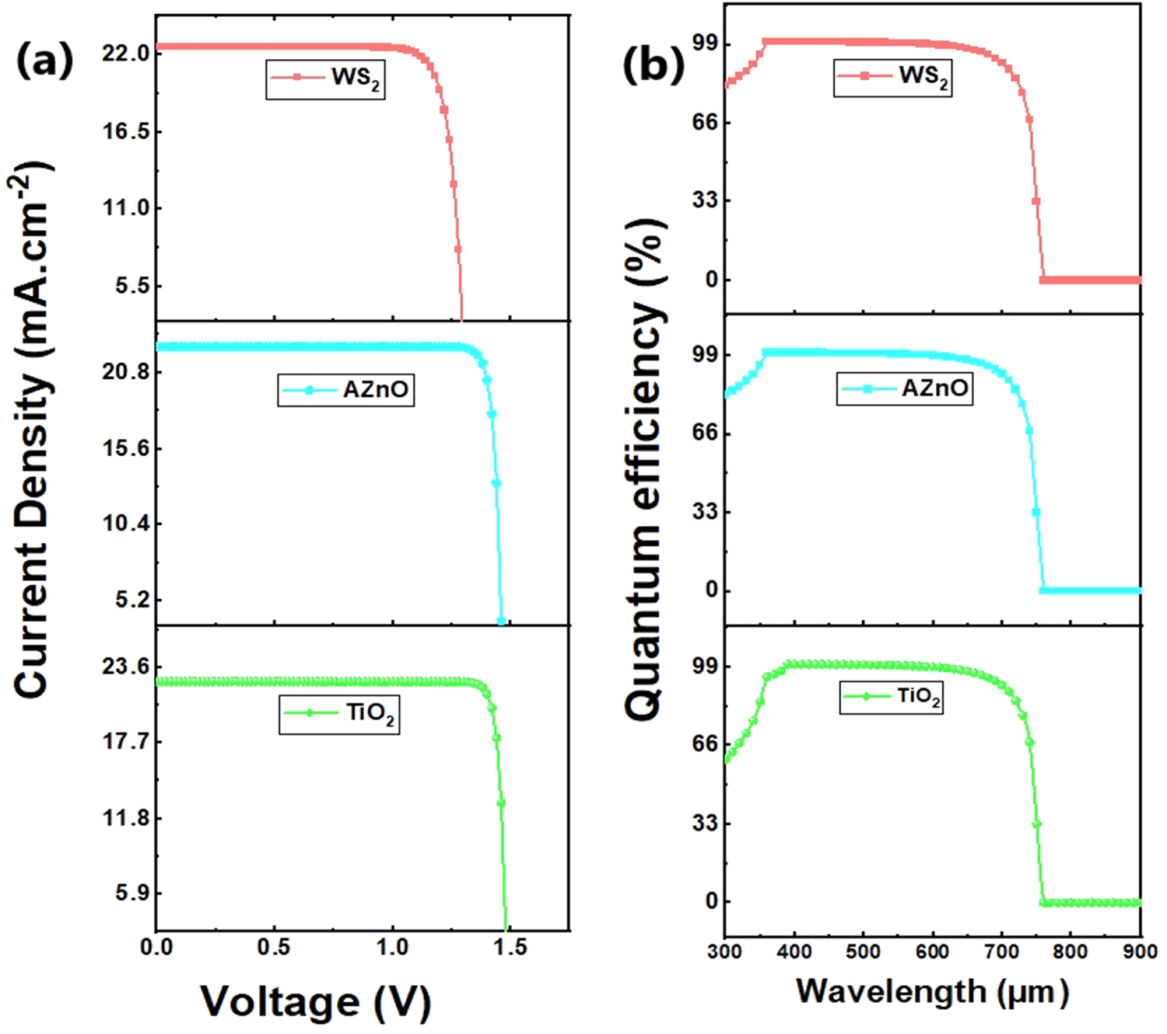
(a) *J*–*V* characteristics and (b) quantum efficiency for every device under study.

The current–voltage characteristics of the simulated devices are illustrated in Fig. 24(a). All three devices exhibited relatively high short-circuit current densities (*J*_SC_ ∼22.49–22.58 mA cm^−2^), indicating that the CaHfS_3_ absorber effectively captures light and generates carriers. However, there are significant differences in overall current stability and open-circuit voltage (*V*_OC_). The cell utilizing AZnO as the ETL maintains a stable current plateau across a wide voltage range and achieves the highest *V*_OC_ (≈1.46 V). This suggests reduced interfacial recombination losses and excellent band alignment at the AZnO/CaHfS_3_ interface, indicating that AZnO is the most effective ETL among those studied. Fig. 24(b) shows how wavelength affects quantum efficiency (QE) for our investigation's three most successful devices. According to the data, AZnO and WS_2_ outperform TiO_2_ in the UV (300–360 nm) region, with QE values exceeding 90% compared to TiO_2_, approximately 60%. This improvement may be attributed to the superior electrical and optical characteristics of AZnO and WS_2_, such as their broad band alignment compatibility with CaHfS_3_ and high optical transparency in the ultraviolet region, resulting in more effective charge carrier extraction in the high-energy area. Within the visible spectrum (360–700 nm), all three ETL designs demonstrate virtually optimal quantum efficiency, with values around 100%. This suggests that the CaHfS_3_ absorber efficiently transforms visible light photons into charge carriers and that the ETLs retain good charge transport with negligible recombination losses throughout this range. However, after 700 nm, the QE starts to drop dramatically for all setups. CaHfS_3_'s bandgap limits its ability to absorb low-energy photons in the near-infrared range, causing a noticeable drop at 770–800 nm.

### 3.13. Comparing SCAPS-1D results with earlier research

A comparison with previously reported perovskite-based devices is summarized in Table 4. The FTO/TiO_2_/CaHfSe_3_/MoO_3_/Au and FTO/AZnO/CaHfSe_3_/MoO_3_/Au devices developed in this study exhibit outstanding photovoltaic performance, with *V*_OC_ ≈ 1.52 V, *J*_SC_ ≈ 23.17–23.23 mA cm^−2^, and PCE values exceeding 32%. The TiO_2_-based structure achieves a slightly higher fill factor (91.41% *vs.* 91.02%) and overall efficiency (32.39% *vs.* 32.20%) compared to the AZnO-based device, reflecting lower resistive losses and more efficient charge extraction. These PCEs are significantly higher than those reported in previous studies, particularly for systems using CaHfSe_3_ as the absorber layer. The top-performing FTO/TiO_2_/CaHfSe_3_/MoO_3_/Au device, achieving 32.39% efficiency, highlights the superior optoelectronic properties of CaHfSe_3_ and its favorable energy alignment with MoO_3_. These results confirm the strong potential of the proposed materials and device architecture for high-performance, lead-free thin-film solar cells.

**Table 4:** Device comparison: *V*_OC_, *J*_SC_, FF, and PCE

Structure	*V*_OC_ (V)	*J*_SC_ (mA cm^−2^)	FF (%)	PCE (%)	Year	Ref.
FTO/TiO_2_/CaHfSe_3_/MoO_3_/Au	1.52	23.169	91.41	32.39	—	—
FTO/AZnO/BaHfSe_3_/MoO_3_/Au	1.52	23.23	91.02	32.20	—	—
FTO/WS_2_/BaHfSe_3_/MoO_3_/Au	1.34	23.22	84.85	29.12	—	—
FTO/TiO_2_/CaHfSe_3_/NiO_2_/Au	1.30	20.62	83.78	22.58	2025	19
FTO/TiO_2_/BaZrS_3_/SpiroOMeTAD/Au	0.70	22.00	79.40	12.42	2023	30
FTO/TiO_2_/CaZrS_3_/CuO	0.60	35.73	80.88	17.53	2024	18

### 3.14. Validation against the ideal cell model

A comparison was made between the simulated photovoltaic properties of CaHfSe_3_-based solar cells and those of an ideal single-junction solar cell. The optimized combination, FTO/TiO_2_/CaHfSe_3_/MoO_3_/Au, attained a remarkable PCE of 32.39%, with a *V*_OC_ of 1.52 V, a *J*_SC_ of 23.17 mA cm^−2^, and an FF of 91.41%. This indicates that the results conform to the theoretical boundaries of an ideal single-junction solar cell.^31^ These results demonstrate the great promise of CaHfSe_3_ as an absorber material for lead-free perovskite solar cells, since they approach the theoretical Shockley Queisser limit.

## 4. Conclusion

This makes our study unique as it intends to examine the photovoltaic characteristics of CaHfSe_3_ absorber material in state-of-the-art solar cells. We explored the potential of devices constructed FTO/TiO_2_, AZnO and WS_2_ layers with CaHfSe_3_/MoO_3_/Au using SCAPS-1D simulations. We then analysed the effects of the absorber thickness, the density of defects in the absorber, carrier concentration in electron & hole transport layers, levels of acceptor doping, *etc.*, and how these factors impact the efficiency of the devices. The effects of *R*_S_ and *R*_SH_, and temperature of operation in addition to other variables explored, were also examined in relation to overall photovoltaic performance. A detailed study of the band structure, carrier generation and recombination rates, *J*–*V* characteristics, and quantum efficiency (QE) was presented herein. The optimized device structure FTO/TiO_2_/CaHfSe_3_/MoO_3_/Au yielded a power conversion efficiency (PCE) of 32.39%, with a *V*_OC_ of 1.52 V, *J*_SC_ of 23.17 mA cm^−2^, and fill factor (FF) of 91.41%. In contrast, the FTO/AZnO/CaHfSe_3_/MoO_3_/Au structure had a PCE of 32.20% with near identical electrical properties. Overall, the results suggest the high optoelectronic quality of CaHfSe_3_, and its compatibility with multiple ETLs. Additionally, cheap substitutes for gold in the back contact were examined, including carbon, with only modest losses in efficiency. Overall, this detailed and systematic work contributes to the understanding of CaHfSe_3_-based perovskite solar cells. It represents a potential pathway towards a lead-free, efficient, low-cost, and sustainable photovoltaic technology, and will support clean energy targets of the future.

## Conflicts of interest

The authors declare no conflict of interest.

## Data Availability

The data will be available from the corresponding author on reasonable request. SCAPS-1D is available from https://scaps.elis.ugent.be/.
